# New suggestions in radical surgical treatment of thromboembolism from the pulmonary artery stump after a Fontan operation

**DOI:** 10.3389/fcvm.2024.1462713

**Published:** 2024-09-05

**Authors:** Dai Dac Tran, Long Hoang Vo, Huy Quang Bui, Thu Hoai Doan, Tien Anh Do, Phuong Hong Do, Linh Phuong Nguyen, Phong Ba Nguyen, Viet Bang Nguyen, Ngoc Minh Le, Huu Cong Nguyen, Thanh Ngoc Le

**Affiliations:** ^1^ Cardiovascular Center, E Hospital, Hanoi, Vietnam; ^2^ Department of Science, Technology, Communication & International Cooperation, E Hospital, Hanoi, Vietnam; ^3^ University of Medicine and Pharmacy, Vietnam National University, Hanoi, Vietnam

**Keywords:** thromboembolism, cerebrovascular event, Fontan operation, pulmonary artery stump, single-ventricle lesions

Thromboembolism from the pulmonary artery stump after a Fontan operation is associated with substantial mortality and morbidity, as evidenced by reports of patients with serious events such as stroke, myocardial infarction, and pulmonary embolism (PE) causing serious life-threatening problems and even death (1). More than half of the patients who suffer a stroke following the Fontan procedure are at risk of death or severe neurological sequelae (2). The rate of thromboembolism reported in patients following Fontan surgery is variable but significant, with a persistent postoperative risk after several years. In addition to the fact that clinical thromboembolism often has disastrous outcomes, a high rate of silent thromboembolic events is an enduring concern for physicians in the current era (1, 3–5).

**Figure 1:**
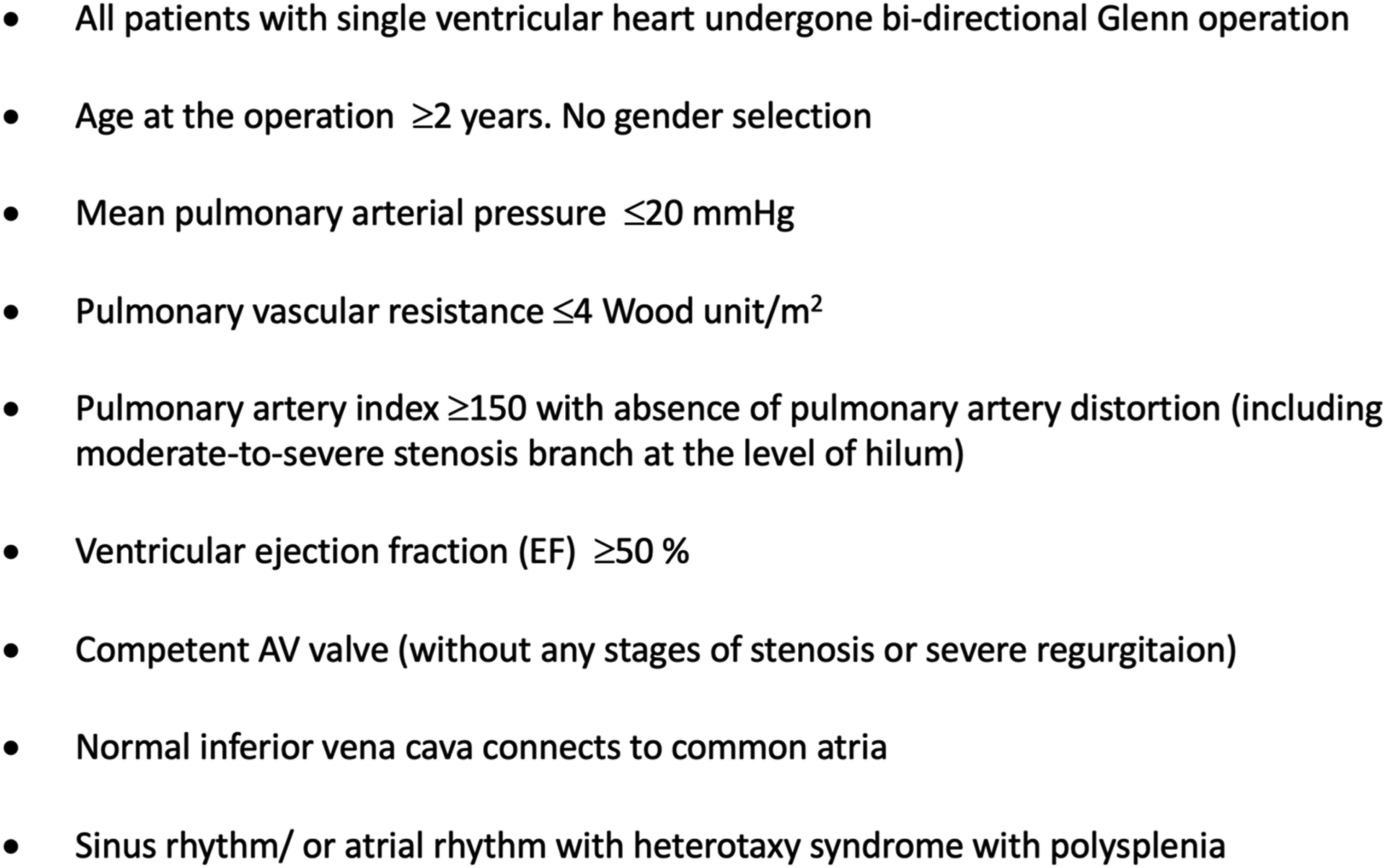
Indications for Fontan surgery.

**Figure 2:**
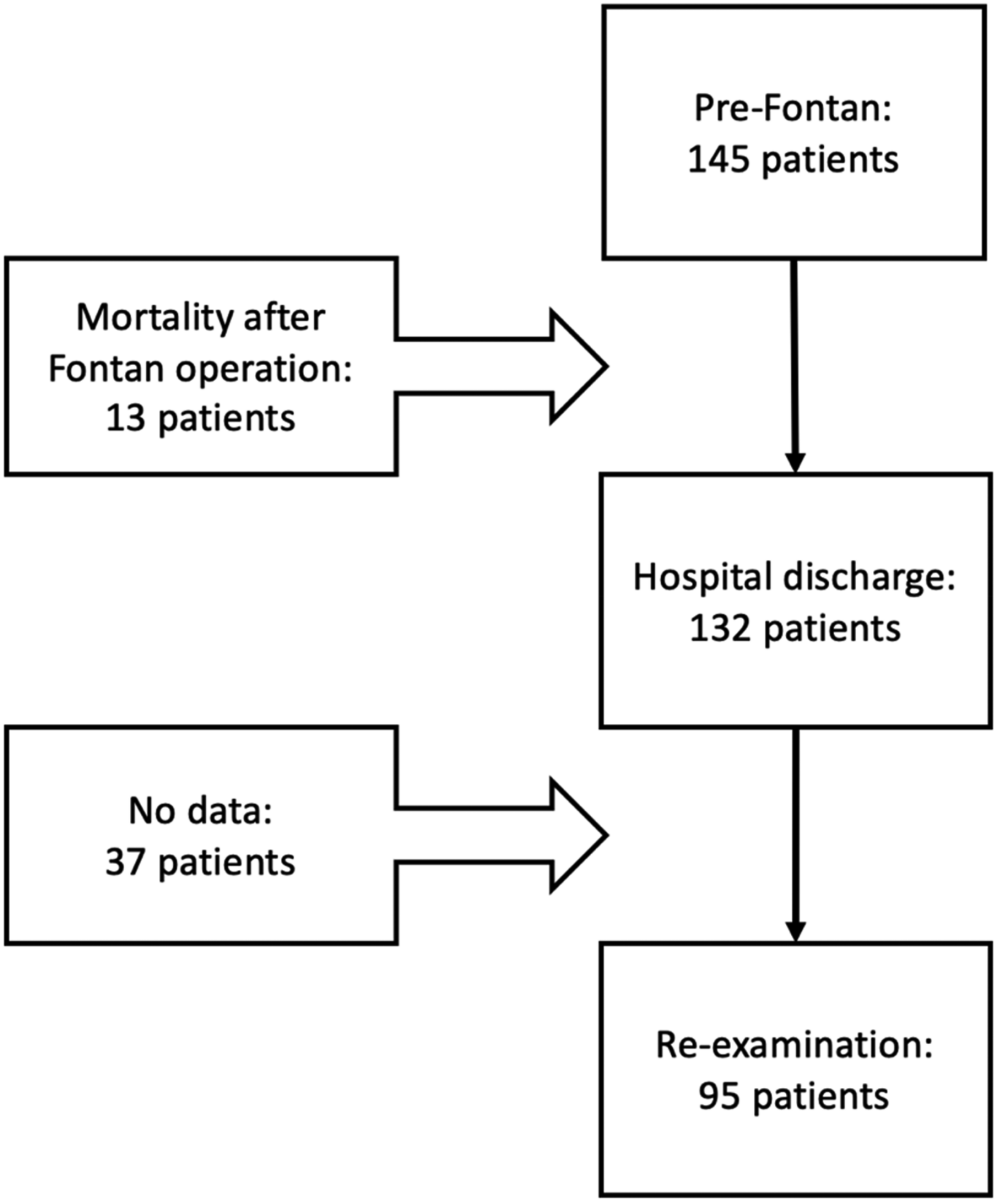
Number of patients undergoing Fontan surgery, discharged after Fontan completion, and during follow-up.

In our study, the institutional surgical database was queried for all children diagnosed with single-ventricle lesions who underwent an extracardiac Fontan operation at the Cardiovascular Center of E Hospital (Hanoi, Vietnam) within an 8-year period. A total of 145 patients underwent a first Fontan operation at E Hospital between 2012 and 2019. Indications for Fontan surgery at our institution are shown in Figure 1. Postoperatively, 132/145 patients were alive at the time of discharge. Of these 132 patients, 95 were re-examined after the Fontan operation, while the data of those remaining could not be documented due to the loss of contact and the disruption of medical visits caused by the COVID-19 pandemic (Figure 2).

**Figure 3:**
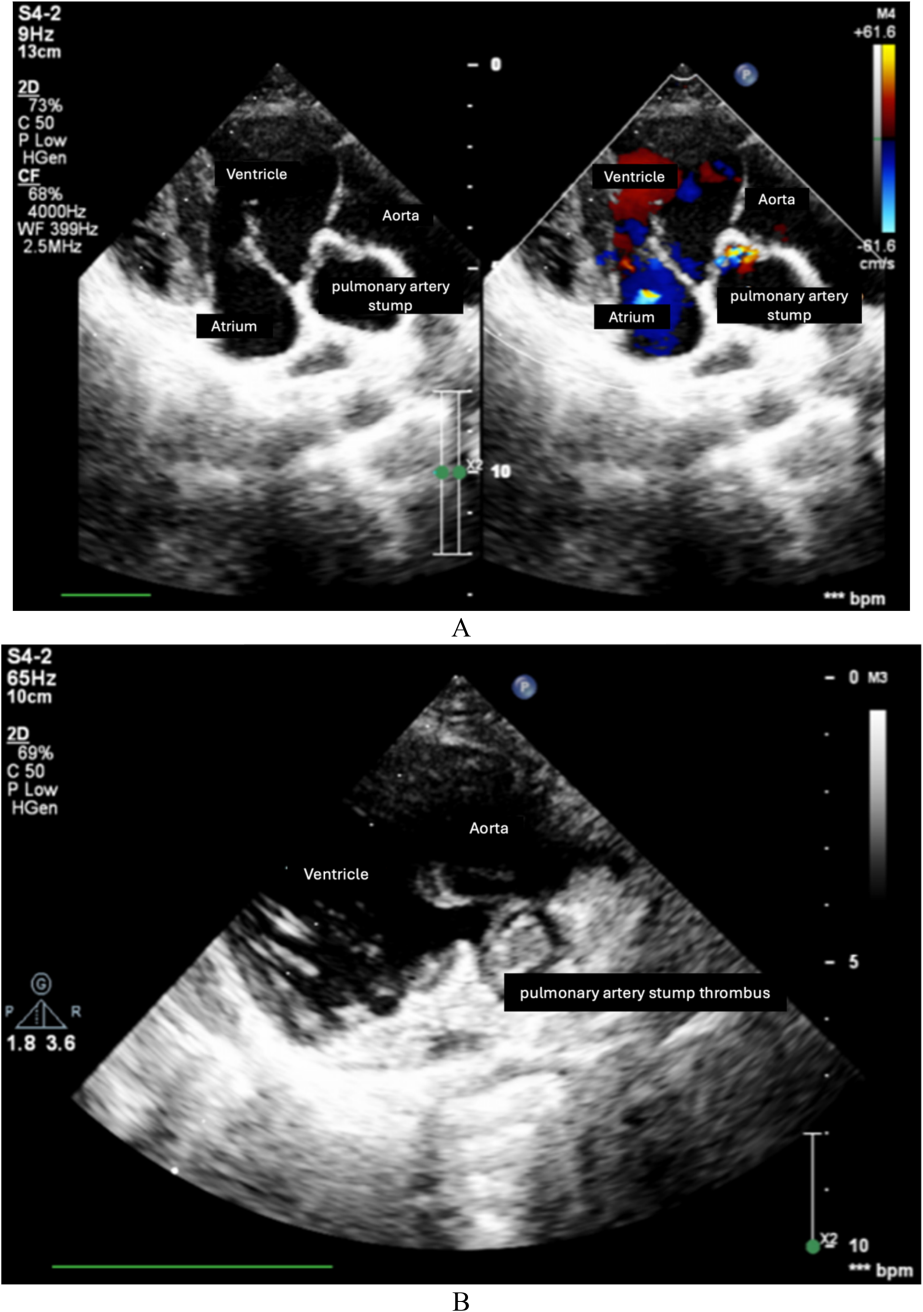
Pulmonary artery stump thrombus and its formation in our case series. **(A)** Pulmonary artery stump between the valve and pulmonary arterial trunk. **(B)** Pulmonary artery stump thrombus. **(C)** Pulmonary arterial trunk closure device after intervention to close the ventricle-to-pulmonary artery shunt. **(D)** After intervention of pulmonary arterial trunk closure with a device.

As was shown in Table 1, echocardiography at hospital discharge of 132 postoperative patients showed that 78 patients (59.1%) had blood flow into the pulmonary artery stump. During the follow-up and re-examination period, 51 out of 95 patients (53.7%) were found to have blood flow at the pulmonary artery stump. Notably, at the time of re-examination, we documented that five patients had thrombus formation occurring at the site of the pulmonary arterial stump (5.3%), even though the patients had been treated with antiplatelet therapy with aspirin. Images of pulmonary artery stump thrombus and its formation in our case series are illustrated in Figure 3. Very few articles to date have indicated thromboembolism from the pulmonary artery stump. We believe that thromboembolism at the site of the pulmonary arterial stump carries a significant risk of cerebral thromboembolism, a rare but serious complication after a Fontan operation. In Koide's early report, there was a case report of a 2-year-old girl who was found to have a large thrombus within the residue of the main pulmonary artery at 3 weeks after the Fontan operation (6). Koide et al. suggested that the thrombus had flowed into the systemic circulation through the ventricular septal defect (6). This patient underwent thrombectomy and pulmonary valve surgical resection 2 weeks after the cerebrovascular event (6).

**Table 1:** Echocardiographic findings of blood flow and thrombus in the pulmonary artery stump at discharge and follow-up.

	Discharge time (*n* = 132)	Follow-up time (*n* = 95)
Blood flow in the pulmonary artery stump (%)		
Yes	78 (59.1)	51 (53.7)
No	54 (40.9)	44 (46.3)
Thrombus in the pulmonary artery stump (%)		
Yes	0 (0.0)	5 (5.3)
No	132 (100.0)	90 (94.7)

**Figure:**
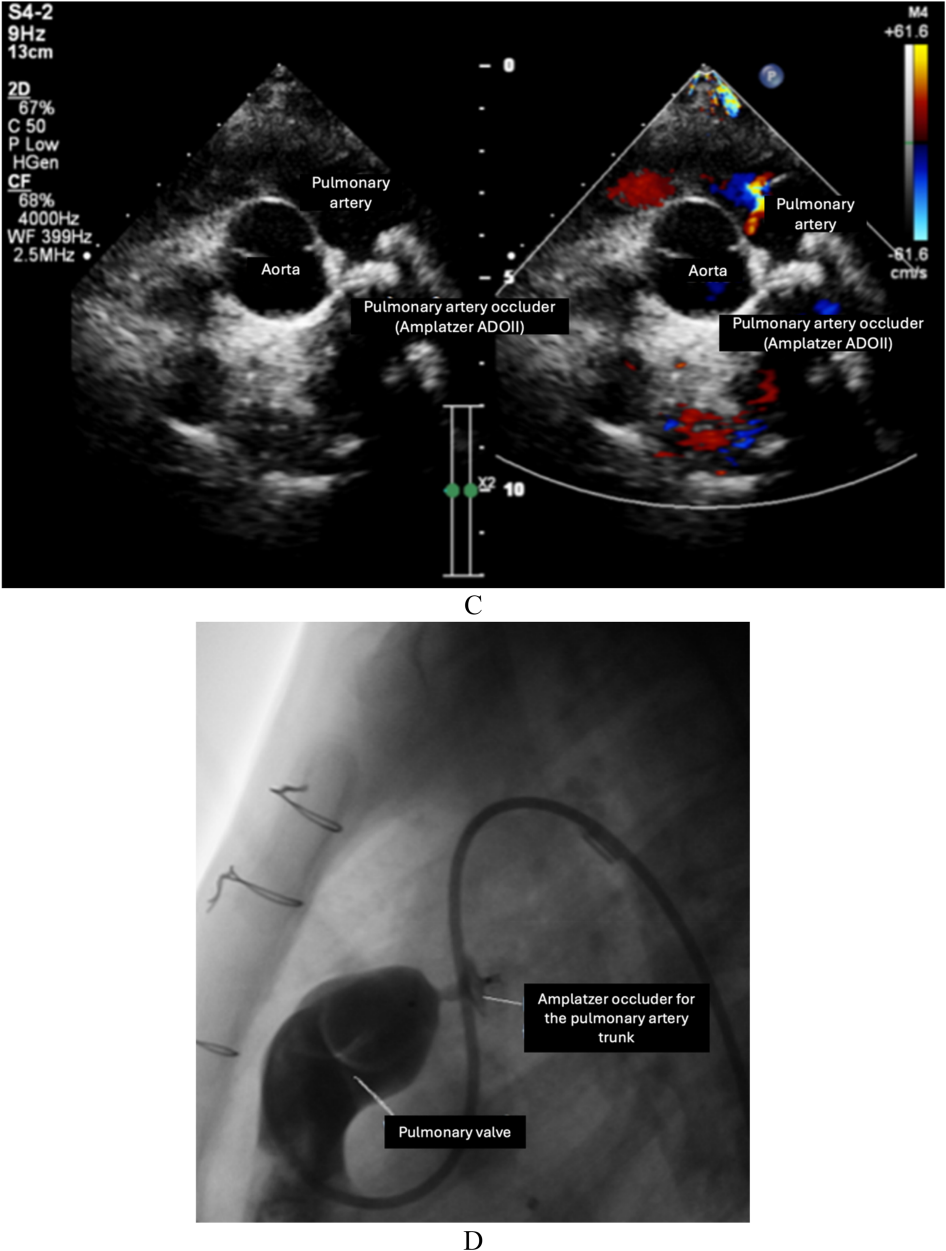


A few cases of cerebral ischemic events after the Fontan operation in children diagnosed with single-ventricle lesions have been documented to date but the incidence is unclear. In a series of patients who had undergone Fontan surgery in our hospital, we had two cases of cerebrovascular accident which caused hemiplegia. In both cases, we found the phenomenon of thrombus formation from the pulmonary artery stump, which was detected after a cerebrovascular accident. This led us to consider the possibility of the thrombus dislodging from the stump, migrating into the ventricle, and subsequently traveling to the brain. These patients had been confirmed to have no residual Fontan fenestration.

One question arises as to whether the thrombus from the pulmonary artery stump moves down the systemic ventricular chamber and moves up to the brain, causing neurological complications. However, the existence of the pulmonary artery stump is also often associated with the existence of a ventricle-to-pulmonary artery shunt. In this study, we occluded the pulmonary trunk in 12 cases at their most recent visit. In patients with single-ventricle heart disease who underwent Fontan surgery, the presence of a pulmonary artery stump has been associated with a higher risk of thromboembolic events, such as stroke, compared to those without a pulmonary artery stump. Studies indicate that the pulmonary artery stump may serve as a nidus for thrombus formation, which can subsequently dislodge and lead to systemic embolization, particularly to the cerebral circulation. Conversely, patients without a pulmonary artery stump generally have a lower incidence of these complications, likely due to the reduced likelihood of thrombus formation in the absence of residual vascular structures. According to the literature, several conditions associated with those with cerebral embolism have been reported to be associated with operations using the fenestrated Fontan procedure or operations with the separation of two branches of the pulmonary artery (7); however, non-fenestrated Fontan existed in our patient cohort. However, in our patient group, the risk of vascular thrombosis was directed to thrombosis at the site of the pulmonary arterial stump. Based on the mechanism of thrombus formation at the pulmonary arterial stump, we propose two specific interventions. Congenital heart surgeons should cut out the entire pulmonary valve loops to create ventilation between the ventricles and the ligation position of the pulmonary trunk so that the blood flow does not become entangled in it, leading to the formation of thrombosis. In addition, performing a closed stitch of the pulmonary valve should also be considered because, as a result, blood flow from the ventricles is then prevented from moving up the pulmonary trunk. In patients with stumps, there may be residual valve tissue or incomplete excision, potentially leading to thrombotic complications. In contrast, in patients without stumps, complete removal or proper management of the valve reduces such risks. The management of the pulmonary artery stump during Fontan surgery is therefore crucial to minimize these risks and improve long-term outcomes in this patient population.

To our knowledge, based on the existing literature and our own clinical observations, the presence of pulmonary artery stumps does not appear to be directly influenced by whether the pulmonary valve is sutured during the dissection. The primary factor contributing to the formation of a stump is the extent and location of the dissection, rather than the specific handling of the pulmonary valve. In particular, when the pulmonary trunk is divided and not properly excised down to the bifurcation, a residual stump can persist irrespective of whether the valve was sutured. However, the state of the pulmonary valve can potentially impact blood flow dynamics and thrombus formation at the stump. For instance, when the valve is not sutured or completely excised, there may be altered hemodynamics that lead to stasis, increasing the risk of thrombus formation within the stump. In contrast, proper excision of the valve and management of the trunk can minimize blood flow disturbances and reduce the risk of thrombosis. In our cohort study, we observed that thrombus formation at the stump was more closely associated with the handling of the pulmonary artery trunk and the management of blood flow, rather than the suturing of the pulmonary valve itself. Nonetheless, we acknowledge that this aspect warrants further investigation to better understand the complex interplay between these surgical factors.

There is currently no evidence supporting the use of anticoagulants following thorough treatment of the pulmonary artery stump. Concern is often raised among clinicians regarding patients with thrombi at the stump, leading to an increased use of anticoagulants, including vitamin K antagonists and antiplatelet agents. During follow-up, there is persistent anxiety about the potential migration of thrombi into the systemic circulation, which could result in significant thromboembolic complications. In contrast, patients without such concerns are typically managed with antiplatelet therapy alone.

**Figure 4:**
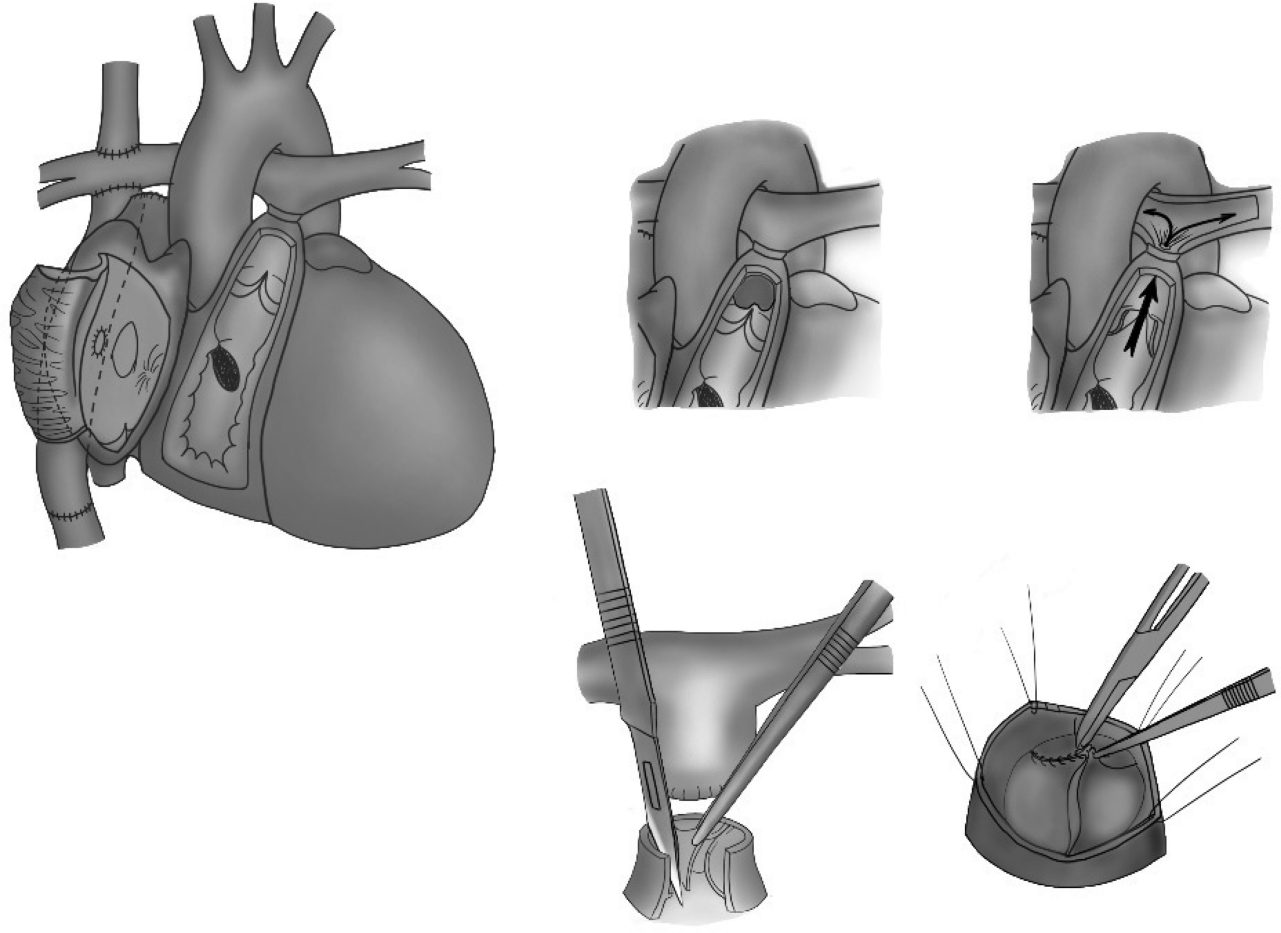
The removal of the entire pulmonary valve to facilitate the flow of blood in and out of the stump or the performance of close sutures at the pulmonary valve.

From our initial experience at a leading Cardiovascular Center, there are two reasons for a thorough treatment of the pulmonary artery stump: preventing thrombus formation and avoiding a residual ventricular-to-pulmonary artery shunt. Specifically, the removal of the entire pulmonary valve should facilitate the flow of blood in and out of the stump, or close sutures should be performed at the pulmonary valve (Figure 4). Further studies are needed to suggest an individual protocol for the complete management of the pulmonary artery stump in patients undergoing the Fontan operation, with the aims of avoiding complications due to stump existence and unnecessary interventions following the Fontan operation.
